# Performance of antigen detection for HRP2-based malaria rapid diagnostic tests in community surveys: Tanzania, July–November 2017

**DOI:** 10.1186/s12936-022-04383-4

**Published:** 2022-12-01

**Authors:** Eric Rogier, Catherine Bakari, Celine I. Mandara, Mercy G. Chiduo, Mateusz Plucinski, Douglas Nace, Nastassia Battle, Franky Chacky, Susan F. Rumisha, Fabrizio Molteni, Renata Mandike, Sigsbert Mkude, Ritha Njau, Ally Mohamed, Venkatachalam Udhayakumar, Deus S. Ishengoma

**Affiliations:** 1 Malaria Branch, Division of Parasitic Diseases and Malaria, Centers for Disease Control and Prevention, Atlanta, GA 30029 USA; 2 National Institute for Medical Research (NIMR), Dar es Salaam, Tanzania; 3 National Institute for Medical Research, Tanga Research Centre, Tanga, Tanzania; 4 CDC Foundation, Atlanta, GA USA; 5 National Malaria Control Programme, Dodoma, Tanzania; 6 Malaria Atlas Project, Geospatial Health and Development, Telethon Kids Institute, Perth, WA Australia; 7 Swiss TPH, Dar es Salaam, Tanzania; 8 World Health Organization Country Office, Dar es Salaam, Tanzania; 9 Harvard T.H Chan School of Public Health, Boston, MA USA; 10 Faculty of Pharmaceutical Sciences, Monash University, Melbourne, Australia

**Keywords:** Tanzania, Malaria, Rapid diagnostic tests, Histidine-rich protein 2, Limit of detection *Plasmodium falciparum*

## Abstract

**Background:**

Malaria rapid diagnostic tests (RDTs) based on the detection of the *Plasmodium falciparum* histidine-rich protein 2 (HRP2) antigen are widely used for detection of active infection with this parasite and are the only practical malaria diagnostic test in some endemic settings. External validation of RDT results from field surveys can confirm appropriate RDT performance.

**Methods:**

A community-based cross-sectional survey was conducted between July and November 2017 enrolling participants of all ages in households from 15 villages in four border regions of Tanzania: Geita, Kigoma, Mtwara and Ruvuma. All participants had an RDT performed in the field and provided a blood sample for later laboratory multiplex antigen detection of HRP2. In assessing the continuous HRP2 levels in participant blood versus RDT result, dose–response logistic regression provided quantitative estimates for HRP2 limit of detection (LOD).

**Results:**

From the 15 study villages, 6941 persons were enrolled that had a RDT at time of enrollment and provided a DBS for later laboratory antigen detection. RDT positive prevalence for the HRP2 band by village ranged from 20.0 to 43.6%, but the magnitude of this prevalence did not have an effect on the estimated LOD of RDTs utilized in different villages. Overall, HRP2 single-target tests had a lower LOD at the 95% probability of positive RDT (4.3 ng/mL; 95% CI 3.4–5.4) when compared to pLDH/HRP2 dual target tests (5.4 ng/mL; 4.5–6.3), though this difference was not significant. With the exception of one village, all other 14 villages (93.3%) showed RDT LOD estimates at 90% probability of positive RDT between 0.5 and 12.0 ng/mL.

**Conclusions:**

Both HRP2-only and pLDH/HRP2 combo RDTs utilized in a 2017 Tanzania cross-sectional survey of border regions generally performed well, and reliably detected HRP2 antigen in the low ng/mL range. Though single target tests had lower levels of HRP2 detection, both tests were within similar ranges among the 15 villages. Comparison of quantitative HRP2 detection limits among study sites can help interpret RDT testing results when generating population prevalence estimates for malaria infection.

**Supplementary Information:**

The online version contains supplementary material available at 10.1186/s12936-022-04383-4.

## Background

Malaria continues to be a significant public health threat worldwide, with sub-Saharan Africa remaining disproportionately affected by the disease. The World Health Organization (WHO) reported an estimated 241 million cases of malaria and 627,000 deaths globally in 2020, of which > 90% of deaths occurred in sub-Saharan Africa [1]. Tanzania, comprised of Mainland and Zanzibar, is counted among the top 10 for highest malaria disease burden out of all endemic countries worldwide in 2020, with an estimated 3% of global cases and 4% of global deaths occurring in that year [1]. *Plasmodium falciparum* accounts for 96% of malaria infections in Tanzania [2], with transmission rates varying significantly both among and within regions across the country [3–5]. Due to recent successes in malaria control [3], the National Malaria Control Programme (NMCP) has resolved to pursue Tanzanian malaria elimination in phases, through standardized epidemiological strata, in the near future. Continued malaria surveillance measures, proper disease diagnosis/treatment, and prevention efforts will be paramount in achieving this elimination goal.

In adherence to the WHO 2010 recommendation that all suspected cases of malaria have parasitological confirmation before treatment [6], Tanzania utilizes microscopy and rapid diagnostic tests (RDTs) in the country's current malaria diagnostic testing strategy [3]. Microscopy is considered the gold standard diagnostic test due to its high degree of specificity and its ability to differentiate *Plasmodium* species. However, microscopy-based diagnosis in malaria-endemic countries can many times present challenges given the need for trained microscopists, quality microscopes, reliable electricity and quality reagents for accurate diagnosis. Malaria rapid diagnostic tests detecting parasite antigen in the blood of infected persons have overcome many of these limitations and are widely used in Tanzania for diagnosis of *P. falciparum* infection [2, 4]. RDTs are a pragmatic and reliable diagnostic tool and can be performed without advanced training, are easily interpretable, do not require electricity, and provide results within 15–30 min [7]. Antigen-based malaria RDTs are currently available to detect three different *Plasmodium* blood stage antigens: aldolase, lactate dehydrogenase (LDH), and histidine-rich protein 2 (HRP2) [8, 9]. However, since *P. falciparum* is the predominant species responsible for symptomatic malaria in sub-Saharan Africa, many countries use RDTs detecting only the HRP2 antigen as this provides a sensitive and species-specific marker for infection with this parasite.[7, 10]. As infected erythrocytes containing *P. falciparum* parasites can sequester in host tissue and vasculature, detection of parasite antigen (such as HRP2) may provide a more accurate representation of true parasite density during infection and has been linked to pyrogenic threshold and disease severity [11, 12]. Tanzania also heavily relies on HRP2-RDTs with about 93% of all malaria diagnosis in 2020 and 2021 performed with this type of test (Daniel Mbwambo, personal communication).

In order to assess ability of RDTs to detect lower-density infections, the WHO recommends that malaria RDTs have a panel detection score of ≥ 75% against a panel of *P. falciparum* parasites which have been diluted to 200 p/µL blood [8]. However, this parasite density does not correspond to a particular HRP2 concentration as differences in *P. falciparum* strains and individual infections lead to a weak correlation between parasite and antigen levels [13–15]. The sensitivity of an RDT at the point-of-care depends on several factors, including storage conditions of the test, accuracy of the testing procedures, and the concentration of the targeted antigen in the sample [10]. For a particular survey, RDT performance can be assessed post hoc if an RDT result and blood sample to measure antigen concentration exist for the same study participant [16].

In this study, the analytical sensitivity of RDTs was assessed across 15 villages in Tanzania from a 2017 survey of border regions with persistently-high malaria burden in the country [17]. Sensitivity of HRP2 detection for HRP2-only and pLDH/HRP2-detecting RDTs were compared to each other, and among regions in Tanzania with high *P. falciparum* transmission intensities.

## Methods

### Ethics

As described previously [17], study and the laboratory analyses obtained ethical approval from the Medical Research Coordinating Committee (MRCC) of the Tanzanian National Institute for Medical Research (NIMR) (reference number NIMR/HQ/R.8a/Vol.IX/2551). Permission to conduct the study in the selected regions was sought from the President’s Office, Regional Administration and Local Government (PO-RALG) authorities and Regional, District and village authorities. Participants informed consent/assent was gained before administering study questionnaire or blood testing and collection. Written informed consent for malaria laboratory analyses was obtained from the sample donors. The laboratory activities undertaken at the US Centers for Disease Control and Prevention (CDC) were considered non-research by the CDC Human Subjects office for the purpose of providing laboratory testing of these specimens and participation of CDC scientists for this collaboration (0900f3eb81a8f1ac).

### Study sites

As described previously [17], samples and survey data were obtained from a cross-sectional community survey enrolling non-treatment seeking individuals and conducted between July and November 2017 in four regions of Tanzania (Geita, Kigoma, Mtwara and Ruvuma). These four regions are among the 7 regions with persistently high malaria burden in the past two decades targeted by the National Malaria Control Programme (NMCP) for reduction of malaria burden through WHO and NMCP revised strategic plan [5]. Eight districts were selected within these four regions: Nyang’hwale and Chato (Geita), Buhigwe and Uvinza (Kigoma), Mtwara DC and Nanyumbu (Mtwara) and Nyasa and Tunduru (Ruvuma). Within each district, two villages were selected for sampling based on the malaria parasite positivity rates as reported from health facility reports, making a total of 16 villages sampled for parasitological survey with RDT, and laboratory antigen data. Due to transcription errors at the study site, data from the village of Nawaje in the Nanyumbu district was unable to be used for this current analytical study.

In each of the sampled villages, a random sample of ≥ 120 households were selected, and all members (regardless of age) were asked to enroll in the survey [17]. Blood samples were collected by finger prick for screening with malaria RDTs, and formation of dried blood spots (DBSs) for later laboratory analysis. The Care Start Malaria HRP2/pLDH (Pf/Pan) COMBO (RMRM-02571, AccessBio, NJ, USA) RDTs were used in Geita and Kigoma regions, and Lundo village of Nyasa District (Ruvuma region). These were depleted in some villages and replaced by the Care Start Malaria HRP2 (Pf) (RMOM-02571CB, AccessBio, NJ, USA) which were more readily available and used in the remaining villages in Ruvuma and all sites in Mtwara region. Dried blood spots (DBS) on filter papers were collected on Whatman 3MM paper (GE Healthcare, PA, USA), dried for two to four hours, and individually packaged in sealable plastic bags with desiccant. DBS were stored at room temperature until shipment to the US CDC in Atlanta, GA. Participants with RDT positive results were treated according to the national guidelines [2].

### Sample processing and laboratory multiplex assay

A single 6 mm punch of each sample was taken and eluted in blocking buffer containing: phosphate buffered saline (PBS, pH 7.2), 0.5% polyvinyl alcohol (Sigma, St. Louis, MO), 0.8% polyvinylpyrrolidine (Sigma), 0.1% casein (ThermoFisher Scientific, Waltham, MA), 0.5% BSA (Sigma), 0.3% Tween-20, 0.05% sodium azide, and 3 µg/mL *Escherichia coli* extract. Elution from filter paper diluted the samples to a 1:20 whole blood.

For detection of *Plasmodium* antigens, DBS samples were screened by a bead-based multiplex antigen assay for the simultaneous detection of *P. falciparum* HRP2 (HRP2), pan-*Plasmodium* aldolase (aldolase), and pan-*Plasmodium* lactate dehydrogenase (pLDH) based on previously described protocol [13, 17]. To convert from assay signal to antigen concentration, equations were derived from standard curves of purified recombinant HRP2 antigen (Type B, Microcoat Biotechnologie GmbH, Bernried, Germany) as described previously [18].

### Data analysis

Local regression (LOESS) and logistic regression dose–response curves comparing RDT result to DBS HRP2 antigen concentration were created in R (version 4.03.3, R Foundation for Statistical Computing, Vienna, Austria) as described previously [16]. Briefly, A logistic regression model was fit to the dose–response data and was used to estimate the limit of detection (LOD) of HRP2 concentrations at which 50, 75, 90, and 95% of the RDTs would be predicted to return a positive result for the HRP2 band. This parametric analysis is able to generate point estimates for each of these concentrations with 95% confidence intervals.

## Results

### RDT and laboratory antigen results by study sites

**Fig. 1:**
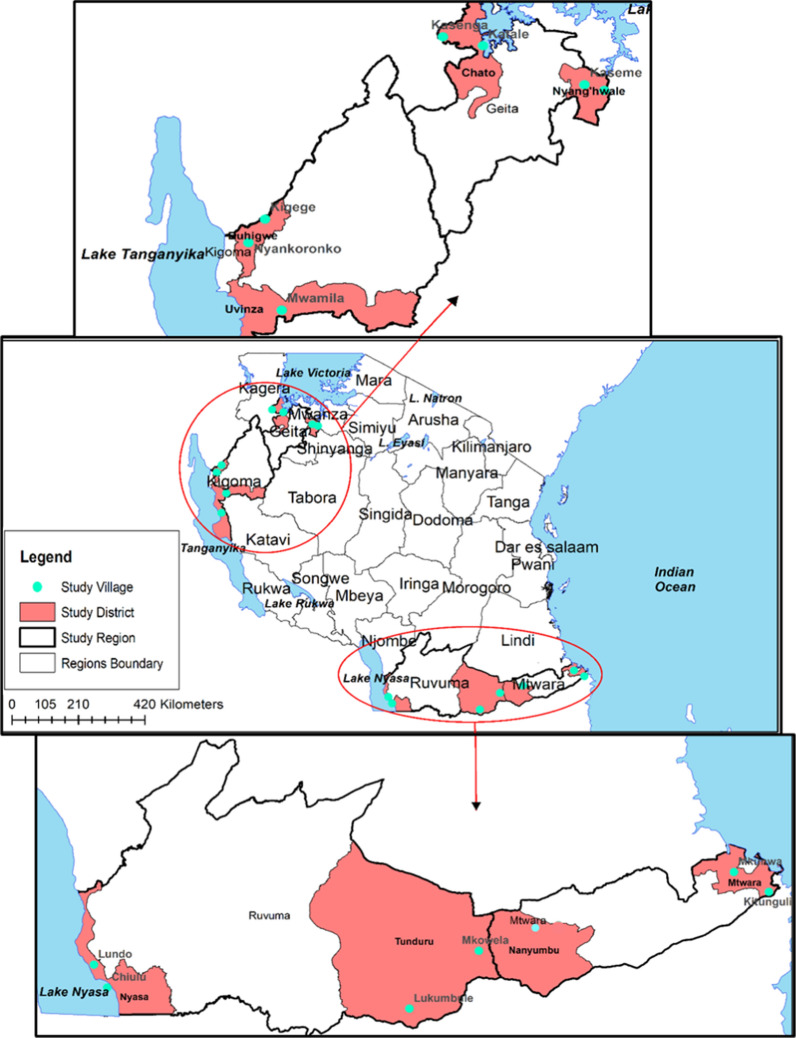
Map showing the study sites—Tanzania cross-sectional survey: July–November 2017. Districts included in this study shaded, and villages for participant enrollment indicated by blue points on the map

In total, 6941 persons had valid RDT results and provided a DBS for later antigen detection by laboratory assay. Of these persons, 2512 (36.2%) were tested with a HRP2-only RDT, and 4429 (63.8%) were tested with a pLDH/HRP2 combo RDT (Table 1). Of 6,941 persons with data for both RDT and HRP2 laboratory assay, 2348 (33.8%) were positive by HRP2 band on the RDT, and 2211 (31.9%) were HRP2 positive by the laboratory assay. There were hundreds of persons enrolled from each of the 15 study villages (map shown in Fig. 1) with Kaseme having the highest number of enrolled participants (545, 7.9% of all) and Mtawatawa having the lowest (397, 5.7%) (Table 2). RDT positivity by village varied from a high of 43.6% in Nyankoronko to a low of 20.0% in Kaseme (Table 2).

**Table 1:** Overall estimates for probability of a positive HRP2 RDT result by levels of HRP2 antigen in blood: Tanzania, 2017

Probability of positive result	CareStart malaria HRP2/pLDH (Pf/Pan) COMBO (n = 4429) (ng/mL, 95% CI)	CareStart malaria HRP2 (Pf) (n = 2512) (ng/mL, 95% CI)
50%	0.93 (0.87–1.0)	0.59 (0.53–0.65)
75%	1.80 (1.6–2.0)	1.25 (1.1–1.4)
90%	3.47 (3.0–4.0)	2.63 (2.1–3.1)
95%	5.44 (4.5–6.3)	4.33 (3.4–5.4)

**Table 2:** Estimated in situ limit of detection of HRP2-based rapid diagnostic tests used as estimated from logistic dose–response model: Tanzania, 2017

Village	Number enrolled(% of all)	RDT type	RDT positive (%)	HRP2 concentration at indicated probability of a positive test (ng/mL, 95% confidence interval)			
				50%	75%	90%	95%
Herembe	473 (6.8%)	pLDH/HRP2	150 (31.7%)	1.53 (1.2–2.1)	4.28 (2.9–6.5)	11.95 (6.6–20)	24.00 (11–42)
Kigege	485 (7.0%)	pLDH/HRP2	166 (34.2%)	1.29 (1–1.7)	2.74 (2–3.8)	5.82 (3.6–8.6)	9.76 (5.3–15)
Kaseme	545 (7.9%)	pLDH/HRP2	109 (20.0%)	1.17 (0.88–1.7)	2.87 (1.8–4.6)	6.96 (3.6–12)	12.71 (5.3–23)
Mwamila	481 (6.9%)	pLDH/HRP2	189 (39.3%)	1.07 (0.85–1.4)	2.29 (1.7–3.2)	4.91 (3.1–7.2)	8.18 (4.6–13)
Kitunguli	399 (5.7%)	HRP2	170 (42.6%)	0.93 (0.63–1.5)	5.96 (3.2–12)	38.41 (14–101)	NA (33.0-NA)
Mkowela	408 (5.9%)	HRP2	159 (39.0%)	0.79 (0.65–0.98)	1.32 (1–1.7)	2.24 (1.5–3.1)	3.19 (2–4.5)
Nyankoronko	532 (7.7%)	pLDH/HRP2	232 (43.6%)	0.78 (0.67–0.95)	1.43 (1.1–1.8)	2.59 (1.8–3.4)	3.88 (2.5–5.3)
Kasenga	474 (6.8%)	pLDH/HRP2	201 (42.4%)	0.78 (0.67–0.93)	1.17 (0.96–1.4)	1.76 (1.3–2.2)	2.33 (1.6–3)
Nyangalamila	489 (7.0%)	pLDH/HRP2	118 (24.1%)	0.68 (0.57–0.86)	1.07 (0.83–1.4)	1.69 (1.1–2.3)	2.30 (1.4–3.2)
Katale	540 (7.8%)	pLDH/HRP2	128 (23.7%)	0.68 (0.56–0.87)	1.09 (0.81–1.5)	1.75 (1.1–2.4)	2.43 (1.4–3.5)
Lundo	410 (5.9%)	pLDH/HRP2	112 (27.3%)	0.65 (0.55–0.78)	0.90 (0.73–1.1)	1.27 (0.94–1.6)	1.60 (1.1–2.1)
Chiulu	435 (6.3%)	HRP2	116 (26.7%)	0.48 (0.42–0.59)	0.69 (0.55–0.86)	0.97 (0.7–1.2)	1.23 (0.81–1.6)
Lukumbule	469 (6.8%)	HRP2	204 (43.5%)	0.42 (0.37–0.48)	0.59 (0.5–0.7)	0.83 (0.65–1.0)	1.05 (0.76–1.3)
Mtawarawa	397 (5.7%)	HRP2	152 (38.3%)	0.26 (0.21–0.33)	0.41 (0.31–0.55)	0.66 (0.43–0.92)	0.92 (0.53–1.3)
Mkunwa	404 (5.8%)	HRP2	142 (35.1%)	0.18 (0.14–0.24)	0.29 (0.21–0.4)	0.48 (0.3–0.7)	0.68 (0.37–1.0)

### Estimates for RDT limit of detection for HRP2 antigen by study site and RDT type

**Fig. 2:**
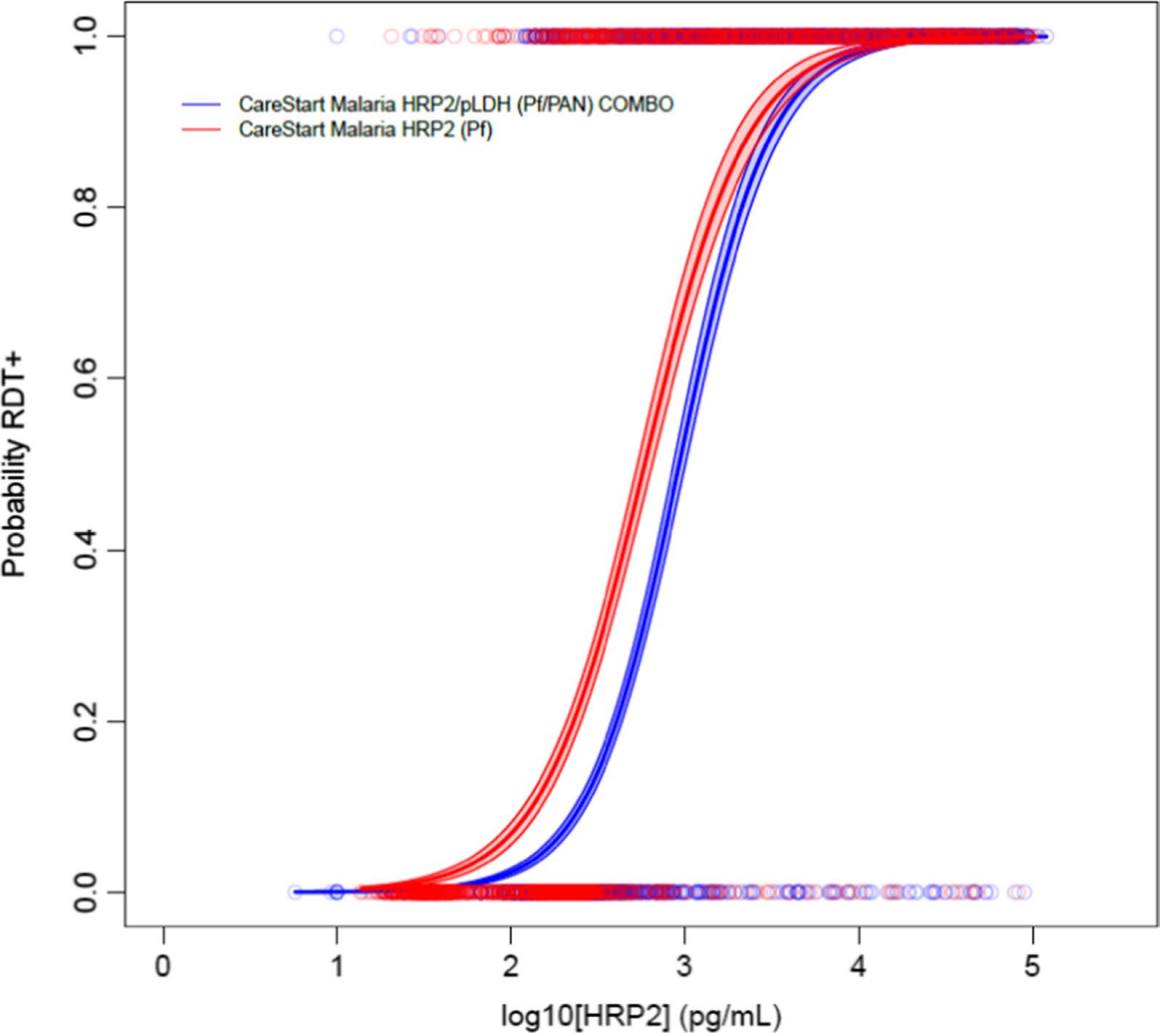
Dose–response logistic and LOESS curves for all sites utilizing either single-target or dual-target RDTs. Comparison of parametric logistic curves between the single-target HRP2-RDT (red dots and red line) and dual-target HRP2/pLDH-RDT (blue dots and blue line) used in this study. Shading on logistic curves indicates 95% confidence limits

**Fig. 3:**
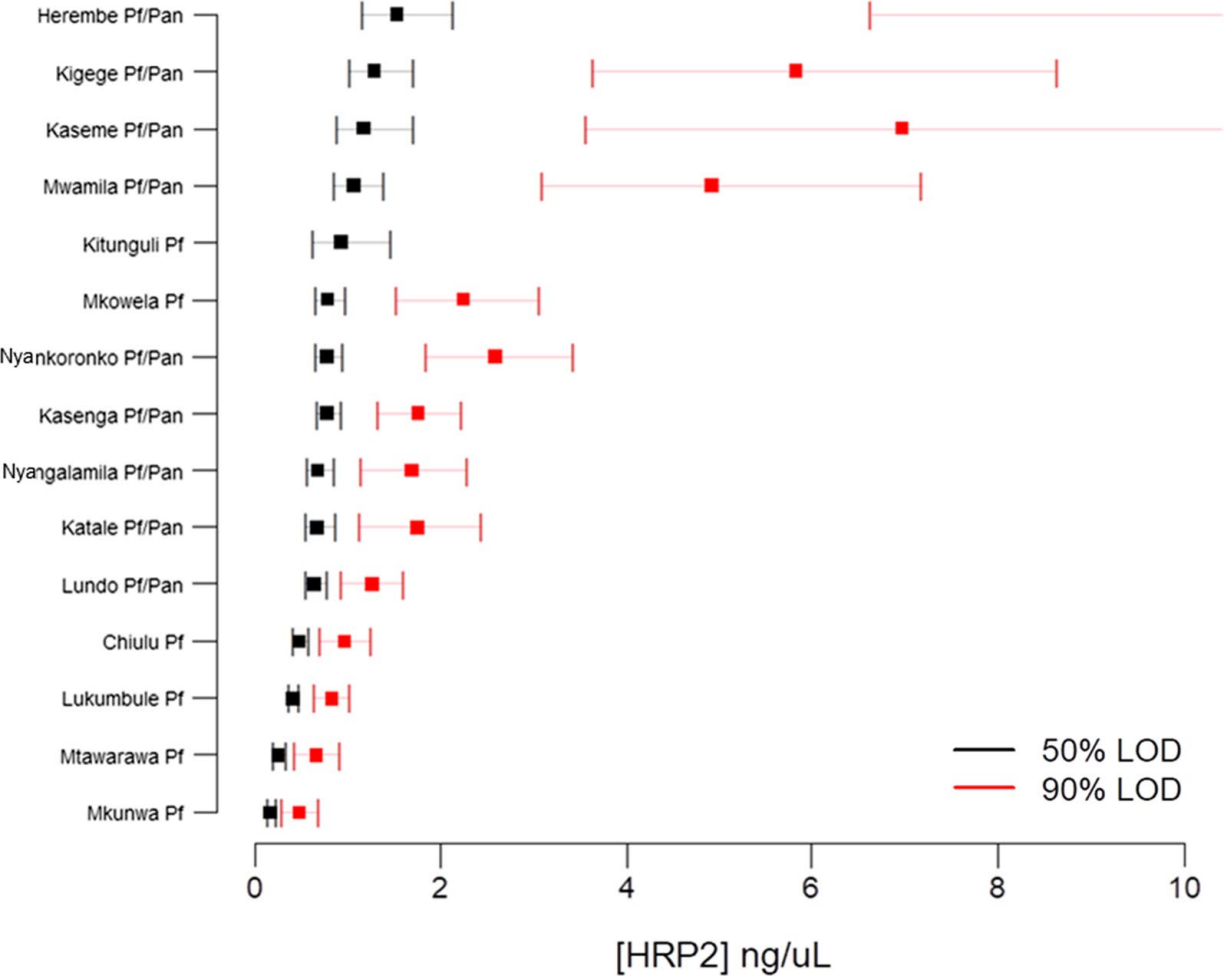
Modelling for RDT detection of HRP2 antigen by village: Tanzania HotSpot study, 2017. Squares represent HRP2 concentration point estimates for 50% (black) and 90% (red) rates of RDT positivity with bars indicating 95% confidence intervals

Overall, the HRP2-only RDT was estimated to detect lower concentrations of HRP2 at all modelled probabilities of RDT positivity when compared to the pLDH/HRP2 RDT (Table 1, Fig. 2). This difference was significant at the 50–75% probability levels (0.59 and 1.25 ng/mL for HRP2-only RDT versus 0.93 and 1.80 ng/mL for the combo RDT), but not significant at the 90% and 95% levels. Overall, RDTs were found to perform well and detect low nanogram ranges of HRP2 antigen in all 15 study villages regardless of brand of RDT used or study population (Table 2, Additional file 1). At a 90% probability of RDT positivity, limit of HRP2 detection ranged from a low of 0.48 ng/mL (95% CI 0.3–0.7) in Mkunwa to a high of 38.4 ng/mL (95% CI 14–101) in Kitunguli (Table 2). Notably, due to the flatter slope of the dose–response curve for Kitunguli (Additional file 1), the 50–75% probability level estimates for this village were very similar to the other villages, but were more inflated at the 90% and 95% levels. With the exception of Kitunguli, the 90% probability level estimates for all other villages were between 0.5 and 12.0 ng/mL (Fig. 3).

### Correlation between *P. falciparum* prevalence and RDT LOD estimates

RDT prevalence by village ranged from 20.0 to 43.6% of all participants testing positive for HRP2 antigen by RDT, indicating a varied range of *P. falciparum* transmission intensities among study sites. When comparing LOD sensitivity estimates for RDT detection of HRP2, no relationship was observed between sensitivity estimates and prevalence of *P. falciparum* infection by percentage of participants RDT positive (Additional file 2). Very low correlation was seen between these two variables with R^2^ values ranging from a low of 0.008 to a high of 0.048, indicating that the modelled probability of RDT positivity estimates were unaffected by population *P. falciparum* infection prevalence. Based on previous estimates of HRP2-based pyrogenic thresholds at ~ 3 ng/mL [11], histograms showed a high proportion of participants who were RDT positive with concentrations estimated to be above this threshold (Additional file 3).

## Discussion

The primary malaria species throughout sub-Saharan Africa is *P. falciparum*, and appropriate and timely diagnosis of this parasite infection is critical for appropriate and effective treatment in clinical settings. Additionally, prevalence results generated through mass testing with RDTs in asymptomatic individuals are increasingly becoming a trusted indicator of malaria transmission in a population regardless of treatment-seeking behaviour [19]. For both clinical and epidemiological purposes, ensuring point-of-contact (POC) RDTs can sensitively detect *Plasmodium* antigens in peripheral blood circulation provides confidence in malaria diagnosis and prevalence estimates. The purpose of this study was to evaluate potential differences in performance of RDTs utilized at different villages from this 2017 Tanzania study, and overall performance of the two different types of RDTs: a RDT only detecting HRP2, and a RDT detecting both pLDH and HRP2. By assaying blood samples with a sensitive HRP2 laboratory assay, this study was able to generate these quantitative RDT performance estimates and found similarities among villages of enrollment and between the two RDT types used.

In order to assess concordance of malaria diagnosis among different tests, many previous studies have compared RDT results to microscopy or nucleic acid-based tests [20–23]. However, the RDT detects parasite antigen, which is inherently different than visual identification of parasites under a microscope or detection of parasite nucleic acids. The most appropriate comparator to POC RDT result is a blood specimen from the same person which is assayed for *Plasmodium* antigens with a more sensitive antigen assay. This has been done successfully many times by post hoc evaluation of RDT performance through quantitative detection of *Plasmodium* antigens by laboratory assays [16, 24–26]. Beyond positive/negative results, RDT analytical sensitivity estimates have also been generated for the current best-in-class RDTs and have estimated reliable HRP2 detection in the range of 1.0 ng/mL [27]. Logistic dose–response modelling of binary RDT result with quantitative HRP2 data can provide predictive estimates (with credible intervals) at which level of *Plasmodium* antigen that a percentage of RDTs (for example, 50% or 95%) from the study would have provided a positive RDT result. This was previously done for a study of febrile patients in Tanzania and showed that the study’s conventional HRP2-based RDT had a reliable sensitivity of approximately 3.7 ng/mL [28]. From this current study, overall LOD estimates were slightly higher for the combo pLDH/HRP2 RDT versus the HRP2-only RDT with the 95% LOD for HRP2 detection at 4.3 ng/mL for the HRP2-only test and 5.4 ng/mL for the combo test. This difference in LOD for the two RDT types was significant at the 50 and 75% levels, but not the 90 and 95% levels. This current estimate for HRP2 detection by a HRP2-only RDT is very similar to the previous Tanzania study of treatment-seeking symptomatic persons (3.7 vs 4.3 ng/mL) [28], and 10 of the 15 villages in this current study had LOD estimates close to what is expected for best-in-class RDTs [27]. Additionally, the finding for slightly lower sensitivity of HRP2 detection for the combo test is also in parallel with what other groups had observed in direct comparisons for single-target and multi-target RDTs [29, 30]. Modification of the RDT test strip to accommodate antibodies to capture and detect multiple *Plasmodium* targets can allow for detection of other *Plasmodium* species (or a redundant test for *P. falciparum*), but this augmented detection capacity inherently leads to a slight reduction in the efficiency of HRP2 binding and detection. Though this current study found HRP2 detection significantly reduced in these combo tests when compared to HRP2-only tests, with such overall low LOD estimates and the low magnitude of percent difference, there is no indication from this data indicating that combo tests are deficient for HRP2 detection.

When comparing LOD estimates by village of enrollment, two prominent findings were observed: more sensitive HRP2 detection in villages only utilizing HRP2-only RDTs, and LOD estimates did not differ by *P. falciparum* prevalence. In this study, RDT prevalence rates ranged from 20.0% to 43.6%, and these results from this community population shows evidence for the high *P. falciparum* transmission in these northwestern and southeastern border regions of Tanzania. These high-transmission regions have been confirmed by recent reports [5, 31, 32], and similar findings by NIMR were the impetus for selection of villages within these border regions for this 2017 survey [17]. No correlation was seen between any RDT LOD estimates (at the 50, 75, 90, or 95% probability levels of RDT positivity) and the observed percentage of the study population infected with *P. falciparum* at the time of enrollment. This finding provides further evidence that the RDTs utilized in this survey performed well in ability to detect persons with HRP2 antigenaemia. Low-density *P. falciparum* infections can still produce nanogram/mL levels of HRP2 in host blood [13], and the HRP2 antigen is known to remain in blood circulation for weeks to months following parasite clearance [33]. For these reasons, higher transmission settings will naturally have a higher frequency of persons with low-levels of HRP2 antigen in their blood. Of the 15 study villages 12 (80%) showed ability to detect HRP2 at less than 10 ng/mL at the 95% probability level of RDT positivity. Of these remaining three, two of those villages had the combo RDT that was utilized which may explain the higher LOD estimates. The consistency in ability of the RDTs to detect low nanogram/mL levels of HRP2 among study villages was supportive evidence for predicting true occurrence of *P. falciparum* infection by the RDT prevalence rates. This endeavour is an important analysis for a study to investigate if there are inherent reasons why study site may have higher (or lower) RDT prevalence estimates, or if significant differences may exist in RDT performance among sites.

This study was also subject to multiple limitations. DBS were prepared in the middle to late 2017, and not processed at CDC until the first half of 2019. This age of specimens combined with storage at ambient temperature would have led to some degree of HRP2 protein degradation, so even though very similar to previous estimates, the LOD estimates presented here are almost certainly an underestimate of the RDTs’ ability to detect the antigen in fresh blood at POC. Additionally, DBS sample storage factors such as humidity and degree of air conditioning could have differed among health facility storage sites and led to varying levels of protein degradation, which would ultimately affect laboratory assay performance and LOD estimates by village. Only two types of RDTs were used in this study, and both from the same manufacturer, so detection of HRP2 by other manufacturers’ products was not able to be assessed here. Unfortunately, lot numbers for the RDTs used in this study were not available, so potential lot-to-lot variation could not be assessed as a potential factor leading to variations in RDT performance through modelled LOD estimates.

## Conclusions

The ability of HRP2-based RDTs to detect low levels of target antigen coupled with the previous finding that *pfhrp2* and *pfhrp3* gene deletions are largely absent in Tanzania [17] provide strong evidence for the continued use of this diagnostic tool for detection of *P. falciparum* infections in the country. As Tanzania works towards malaria control and elimination, continuous evaluation of the performance of this tool (and prevalence of gene deletions) will be critical to ensure the tests are truly capturing infections with this parasite. Moreover, as RDT estimates generated from surveys of non-healthcare settings are increasingly being used for evaluating *P. falciparum* prevalence in many countries, similar data collection and analyses as performed here can provide quantitative estimates for RDT performance among mostly low-density infections.

## Supplementary Information

**Additional file 1**: Dose-response logistic modeling for limits of HRP2 antigen detection by RDT utilized for different study populations. Logistic (red curves) and LOESS (blue curves) regression of probability of RDT positivity by antigen concentration in study participants by village. Shading indicates 95% confidence limits for regression curves. Regression outputs shown in Table 2.

**Additional file 2**: Comparison of village specific RDT prevalence to HRP2 antigen concentration at different probabilities of RDT positivity. Plots shown for estimates for HRP2 concentrations by 50, 75, 90, and 95% probability of positive RDT result with dashed line the linear line of best fit and model estimates. The 95% probability plot does not include Kitunguli village as that point estimate was unable to be calculated.

**Additional file 3**: Antigen concentration versus RDT result in comparison with estimated pyrogenic threshold. Histograms of log-transformed HRP2 concentration shown for each of the village enrolment sites with green bars indicating specimens from RDT-positive persons and blue bars from RDT-negative persons. Grey shading on each plot indicates HRP2 concentrations previously estimated as the pyrogenic threshold for this antigen at >3000 pg/mL (3 ng/mL) (11), and hashed vertical lines for each plot indicate 50% reliability estimates for HRP2 limit of detection (LOD) for each study site.

## Data Availability

Data and non-commercial materials are available upon reasonable request to the corresponding author.

## Abbreviations

CDC: U.S. centers for disease control and prevention
DBS: Dried blood spot
DNA: Deoxyribonucleic acid
HRP2: Histidine-rich protein 2
LOD: Limit of detection
NIMR: Tanzanian national institute for medical research
PBS: Phosphate buffered saline
pLDH: pan-*Plasmodium* lactate dehydrogenase
POC: Point of contact
RDT: Rapid diagnostic test
