# Supplementary material for: Performance of antigen detection for HRP2-based malaria rapid diagnostic tests in community surveys: Tanzania, July–November 2017
Source: Malar J. 2022 Dec 1;21:361. doi: 10.1186/s12936-022-04383-4 (PMC9714097; doi:10.1186/s12936-022-04383-4)
Supplement: Supplementary file 1 — Additional file 1: Dose-response logistic modeling for limits of HRP2 antigen detection by RDT utilized for different study populations. Logistic (red curves) and LOESS (blue curves) regression of probability of RDT positivity by antigen concentration in study participants by village. Shading indicates 95% confidence limits for regression curves. Regression outputs shown in Table 2. [file 12936_2022_4383_MOESM1_ESM.docx]

**Additional Files**

**
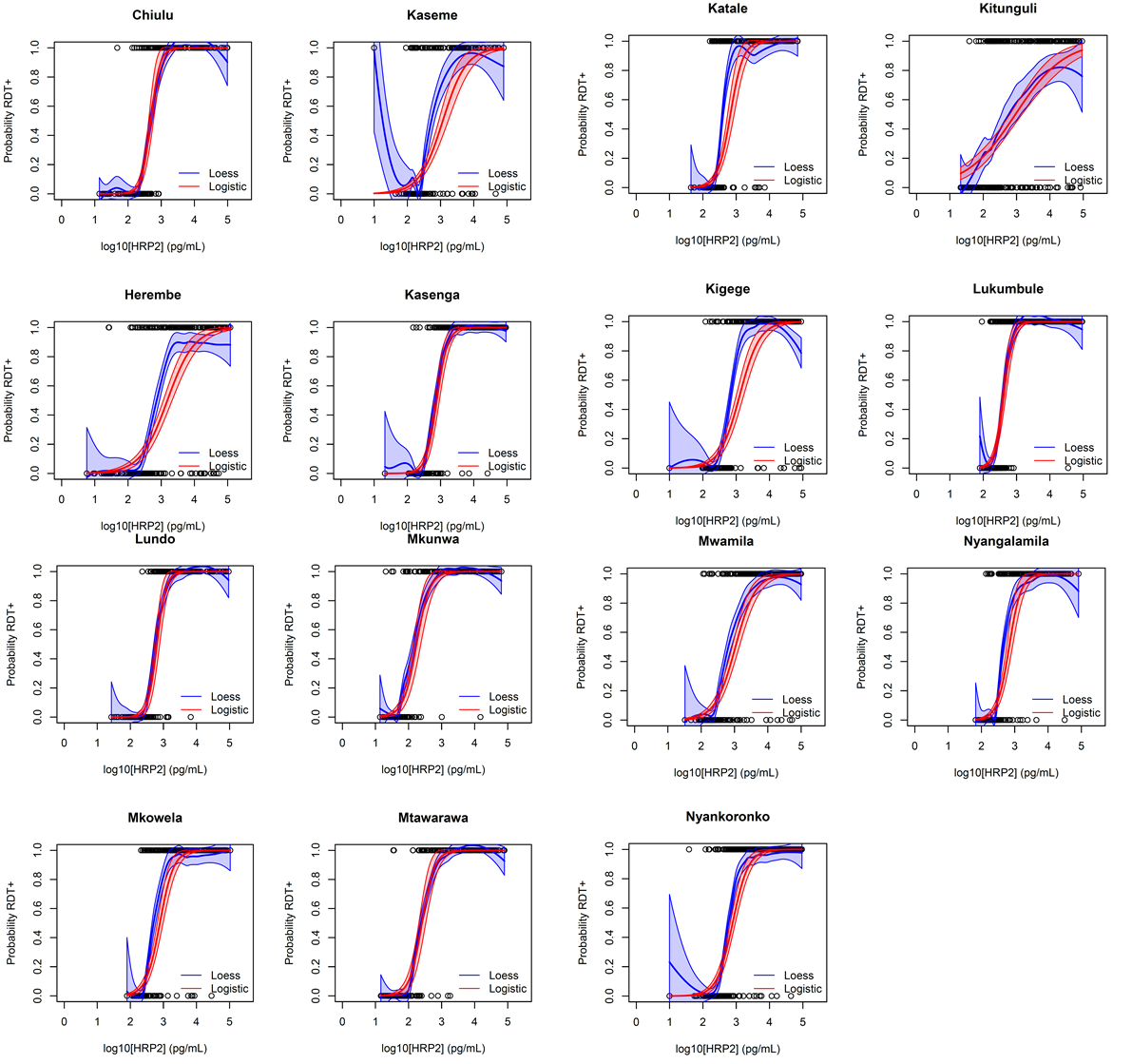
**

**Additional File 1. Dose-response logistic modeling for limits of HRP2 antigen detection by RDT utilized for different study populations**. Logistic (red curves) and LOESS (blue curves) regression of probability of RDT positivity by antigen concentration in study participants by village. Shading indicates 95% confidence limits for regression curves. Regression outputs shown in Table 2.


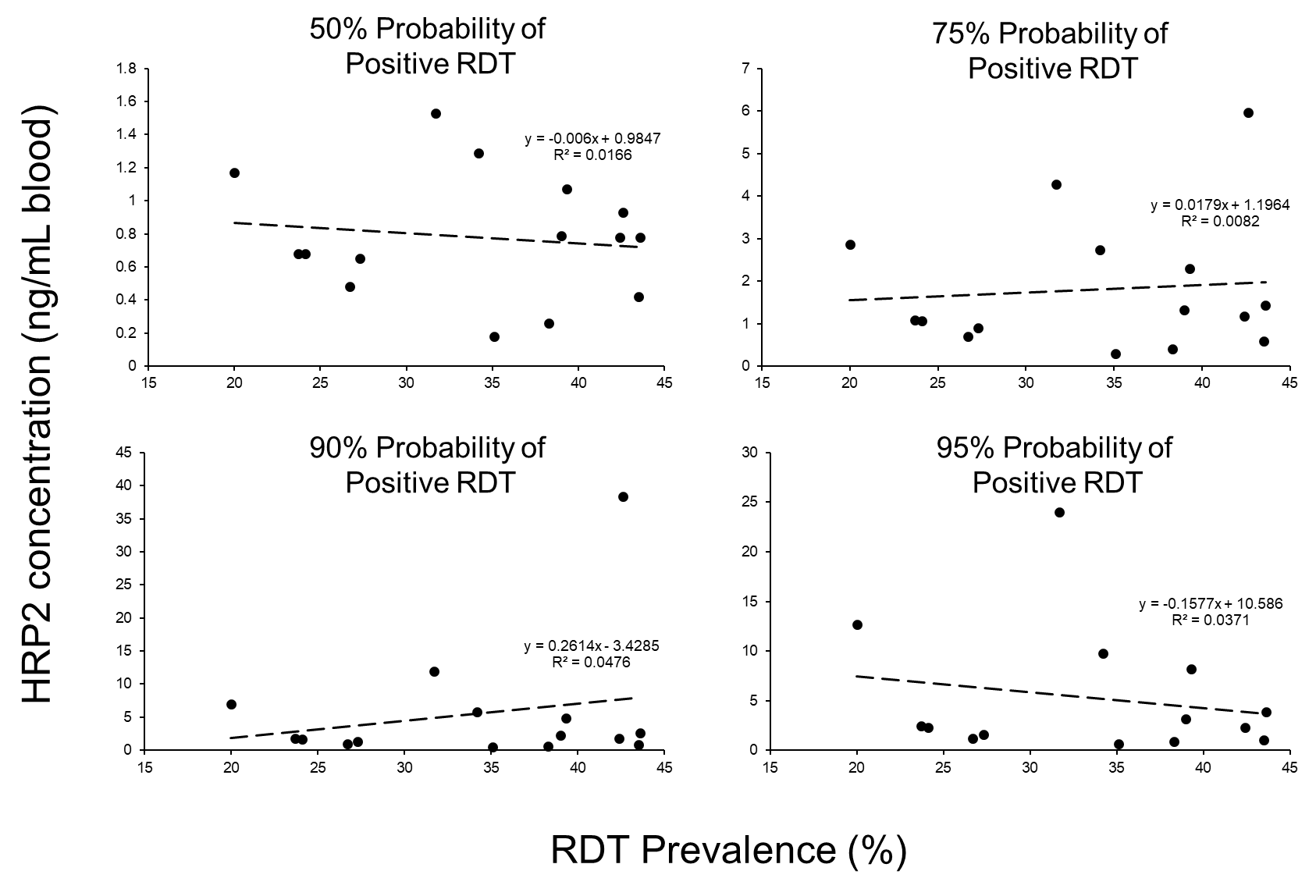


**Additional File 2. Comparison of village specific RDT prevalence to HRP2 antigen concentration at different probabilities of RDT positivity.** Plots shown for estimates for HRP2 concentrations by 50, 75, 90, and 95% probability of positive RDT result with dashed line the linear line of best fit and model estimates. The 95% probability plot does not include Kitunguli village as that point estimate was unable to be calculated.


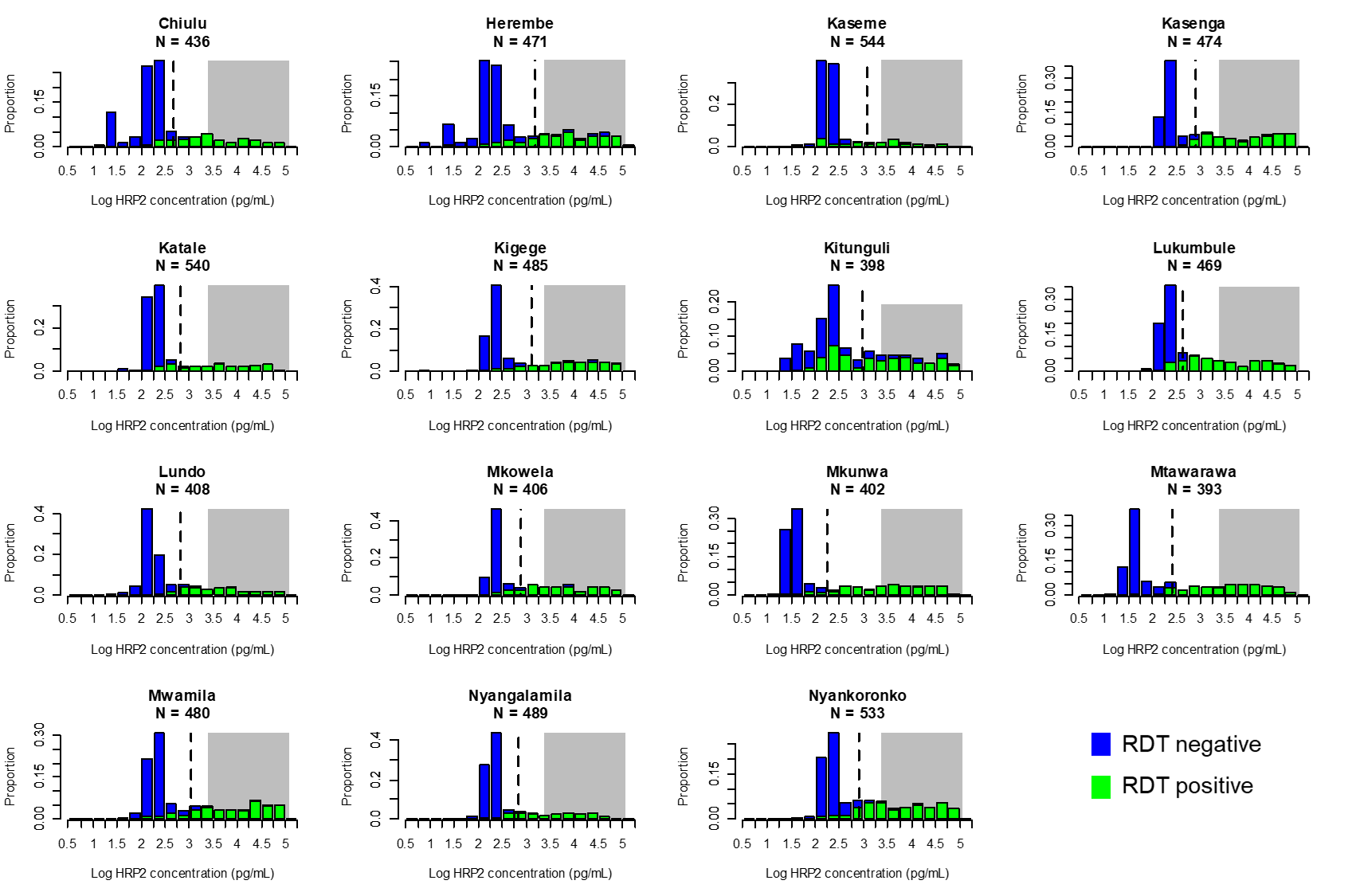


**Additional File 3. Antigen concentration versus RDT result in comparison with estimated pyrogenic threshold.** Histograms of log-transformed HRP2 concentration shown for each of the village enrolment sites with green bars indicating specimens from RDT-positive persons and blue bars from RDT-negative persons. Grey shading on each plot indicates HRP2 concentrations previously estimated as the pyrogenic threshold for this antigen at >3,000 pg/mL (3 ng/mL) (11), and hashed vertical lines for each plot indicate 50% reliability estimates for HRP2 limit of detection (LOD) for each study site.
