# Supplementary material for: Performance of antigen detection for HRP2-based malaria rapid diagnostic tests in community surveys: Tanzania, July–November 2017
Source: Malar J. 2022 Dec 1;21:361. doi: 10.1186/s12936-022-04383-4 (PMC9714097; doi:10.1186/s12936-022-04383-4)
Supplement: Supplementary file 2 — Additional file 2: Comparison of village specific RDT prevalence to HRP2 antigen concentration at different probabilities of RDT positivity. Plots shown for estimates for HRP2 concentrations by 50, 75, 90, and 95% probability of positive RDT result with dashed line the linear line of best fit and model estimates. The 95% probability plot does not include Kitunguli village as that point estimate was unable to be calculated. [file 12936_2022_4383_MOESM2_ESM.docx]

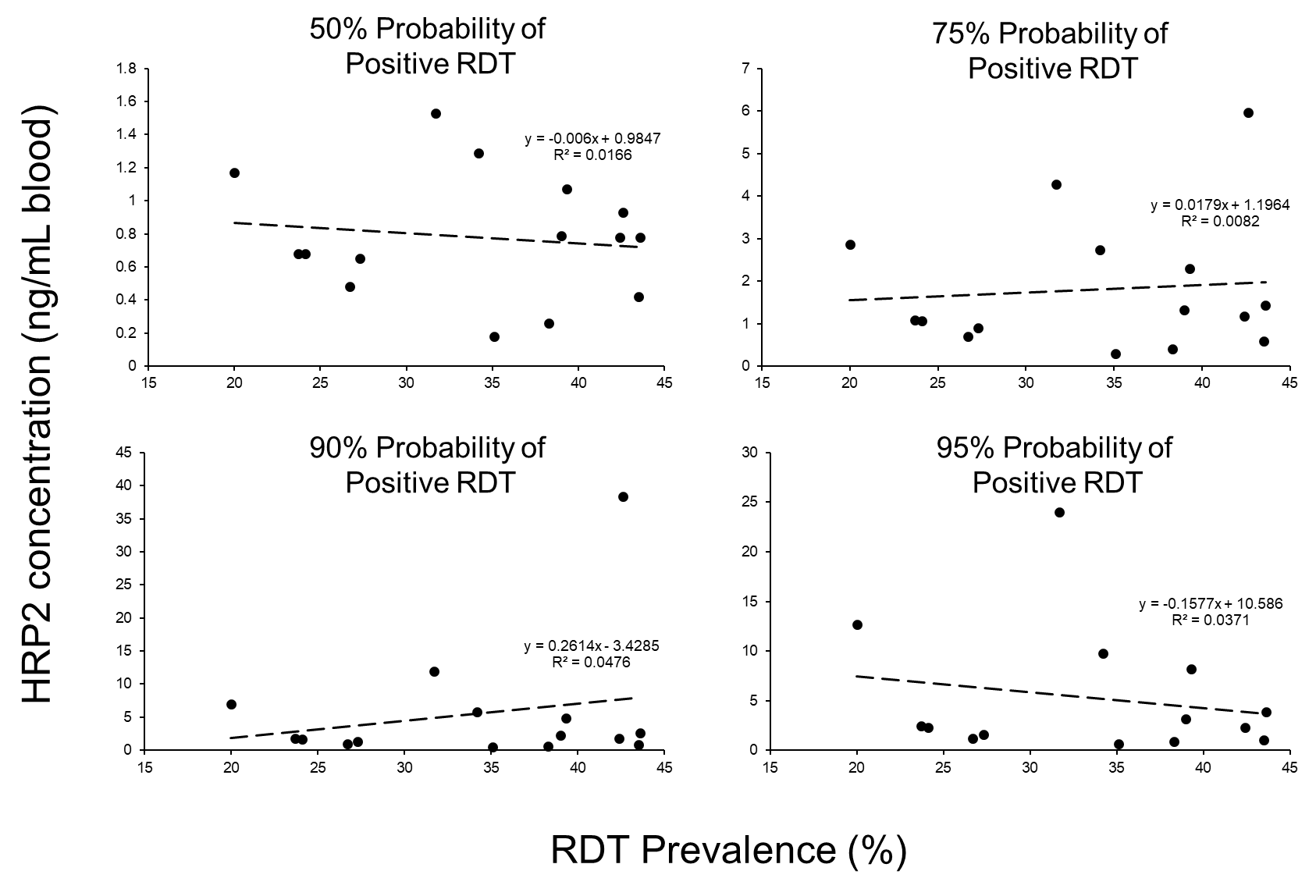


**Additional File 2. Comparison of village specific RDT prevalence to HRP2 antigen concentration at different probabilities of RDT positivity.** Plots shown for estimates for HRP2 concentrations by 50, 75, 90, and 95% probability of positive RDT result with dashed line the linear line of best fit and model estimates. The 95% probability plot does not include Kitunguli village as that point estimate was unable to be calculated.
