# Supplementary material for: Performance of antigen detection for HRP2-based malaria rapid diagnostic tests in community surveys: Tanzania, July–November 2017
Source: Malar J. 2022 Dec 1;21:361. doi: 10.1186/s12936-022-04383-4 (PMC9714097; doi:10.1186/s12936-022-04383-4)
Supplement: Supplementary file 3 — Additional file 3: Antigen concentration versus RDT result in comparison with estimated pyrogenic threshold. Histograms of log-transformed HRP2 concentration shown for each of the village enrolment sites with green bars indicating specimens from RDT-positive persons and blue bars from RDT-negative persons. Grey shading on each plot indicates HRP2 concentrations previously estimated as the pyrogenic threshold for this antigen at >3000 pg/mL (3 ng/mL) (11), and hashed vertical lines for each plot indicate 50% reliability estimates for HRP2 limit of detection (LOD) for each study site. [file 12936_2022_4383_MOESM3_ESM.docx]

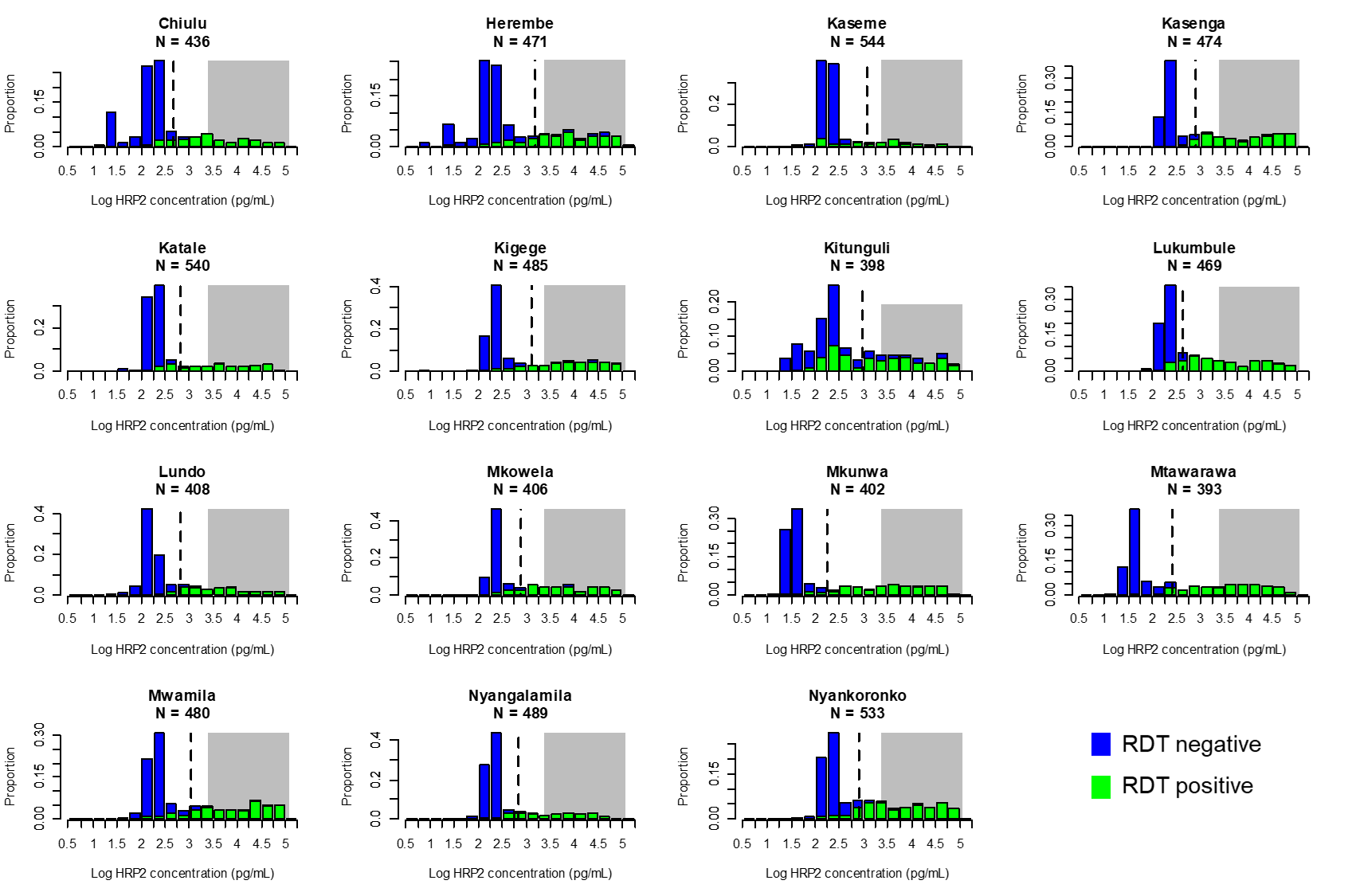


**Additional File 3. Antigen concentration versus RDT result in comparison with estimated pyrogenic threshold.** Histograms of log-transformed HRP2 concentration shown for each of the village enrolment sites with green bars indicating specimens from RDT-positive persons and blue bars from RDT-negative persons. Grey shading on each plot indicates HRP2 concentrations previously estimated as the pyrogenic threshold for this antigen at >3,000 pg/mL (3 ng/mL) (11), and hashed vertical lines for each plot indicate 50% reliability estimates for HRP2 limit of detection (LOD) for each study site.
